# Role of nucleoporins in nuclear import and 3D location of HIV-1 in the nuclear compartment

**DOI:** 10.1186/1742-4690-10-S1-P53

**Published:** 2013-09-19

**Authors:** Ruben Maeso, Francis Ashwanth, Anna Cereseto

**Affiliations:** 1 Centre for Integrative Biology (CIBIO), University of Trento, 38123 Mattarello, Italy

## Background

Emerging evidence suggests a fundamental role of nucleoporins (Nups) during HIV-1 replication. The involvement of Nups in HIV-1 life cycle was demonstrated both through genome-wide siRNA screens and detailed molecular analyses. Nevertheless, the interaction of HIV-1 with Nups has been poorly described: the impact of Nups during viral import through the nuclear pore complex (NPC) and during subsequent events within the nuclear compartment still remains unclear. The aim of this study is to identify the role of specific Nups in the nuclear import of HIV pre-integration complexes (PICs) and during downstream events leading to integration in specific regions of the cellular chromatin.

## Materials and methods

The nuclear import of the PICs was evaluated in Nup depleted cells using an imaging technique which provides a direct visualization of viruses inside the cells. Knock-down cells were generated though silencing technology (siRNA and shRNA). After silencing, the cells were infected with HIV-IN-EGFP viruses carrying integrase fused to EGFP [1]. The comparative analysis of intra-nuclear fluorescent viruses in Nup depleted cells allowed us to determine the implication of Nups in HIV-1 nuclear import.

## Results

Here we demonstrate that selective depletion of nucleoporins 153 and 98, which led to considerable reduction in HIV-1 infectivity, determines an impairment in nuclear PICs trafficking though the NPCs. Besides controlling nucleo-cytoplasmic exchange, several Nups might dynamically interact with PICs away from the NPC. The interaction between HIV and Nups may regulate the HIV-1 events downstream nuclear import. Therefore, we exploited the single-cell imaging of HIV-1 provirus (SCIP) technique, recently set up in our lab [2], to determine the putative role of Nups in integration by 3D analysis of these spots.

## Conclusions

HIV visualization technique used in this study provides a direct measurement of the nuclear import of PICs as opposed to indirect 2-LTR circles measurement. The use of this direct assay coupled with SCIP technique might help us to improve our knowledge on the involvement of Nups in the nuclear import of HIV and in subsequent events within the nuclear compartment.
